# Prevalence and risk factors of depression, anxiety, and stress among secondary school students in Abha city, Saudi Arabia

**DOI:** 10.3389/fpsyt.2026.1885953

**Published:** 2026-08-21

**Authors:** Mohammed A. Jabri, Ibrahim Elsubai, Taqeyah Alshanqiti, Najla Muteb Alotaibi, Maryam Aljohani, Jana Alhazmi, Redwan Mulla, Sadeem Alhejaili, Shatha Althubyani, Majd Nasser Bin Jaber, Shahad Abdullatif Alharbi, Abdulmajeed Sulaiman Alahmadi

**Affiliations:** 1 Preventive Medicine Department, Ministry of Health, Abha, Saudi Arabia; 2 Preventive Medicine Department, Aseer Health Cluster, Abha, Saudi Arabia; 3 Preventive Medicine Department, College of Medicine, Vision College, Riyadh, Saudi Arabia; 4 Preventive Medicine Department, College of Medicine, Taibah University, Madinah, Saudi Arabia

**Keywords:** adolescents, anxiety, DASS-42, depression, mental health, psychological distress, Saudi Arabia, secondary school students

## Abstract

**Background:**

Adolescence is a vulnerable developmental stage associated with an increased risk of depression, anxiety, and stress, which can adversely affect academic performance and quality of life. Despite growing concern about adolescent mental health in Saudi Arabia, evidence among secondary school students remains limited. This study assessed the prevalence and severity of depression, anxiety, and stress among secondary school students in Abha City and identified associated sociodemographic and family-related factors.

**Methods:**

A cross-sectional analytical study was conducted among 400 male and female secondary school students from governmental schools in Abha City, Saudi Arabia, between October and December 2025. Participants were selected using multistage stratified random sampling. Data were collected using a structured questionnaire and the validated Arabic version of the Depression, Anxiety, and Stress Scale (DASS-42). Descriptive statistics, chi-square tests, and multivariable logistic regression analyses were performed using R version 4.4.3, with statistical significance set at *p* < 0.05.

**Results:**

The prevalence of depression, anxiety, and stress was 39.0%, 53.5%, and 38.3%, respectively. Moderate-to-severe symptoms were observed in a substantial proportion of students. Female students had significantly higher rates of all three outcomes (*p* < 0.001). Multivariable logistic regression identified female sex and smoking as independent predictors of depression, anxiety, and stress. Chronic disease and a positive family history of psychiatric disorders were independently associated with depression and stress, whereas increasing family size was associated with lower odds of stress. Maternal employment was independently associated with anxiety, whereas parents not living together were independently associated with depression.

**Conclusion:**

Secondary school students in Abha City experience a high burden of depression, anxiety, and stress, with anxiety being the most prevalent condition. Female sex, smoking, chronic disease, family history of psychiatric disorders, family size, maternal employment, and parental marital status were independently associated with psychological distress. These findings support routine school-based mental health screening, early identification of high-risk students, and targeted psychological support and health promotion programs.

## 1 Introduction

Mental health disorders are among the leading causes of disability worldwide, particularly among adolescents and young adults. Globally, one in seven adolescents aged 10–19 years experiences a mental disorder, accounting for approximately 15% of the global burden of disease in this age group (1). Depression, anxiety, and stress are common mental disorders that adversely affect emotional well-being, academic performance, interpersonal relationships, and quality of life. The World Health Organization (WHO) recognizes mental health as a fundamental component of overall health and emphasizes the importance of early identification and intervention to reduce the long-term consequences of psychological disorders (1).

Adolescence, defined by the WHO as the period between 10 and 19 years of age, is a critical developmental stage characterized by substantial biological, psychological, cognitive, and social changes (2). During this period, adolescents become increasingly sensitive to peer influence, academic demands, and environmental stressors while coping abilities and emotional regulation are still developing (3, 4). According to Bronfenbrenner’s Ecological Systems Theory, adolescent mental health is shaped by interactions between individual characteristics and surrounding environments, particularly the family and school settings, which together influence psychological well-being (5).

Untreated emotional disorders during adolescence may impair educational attainment, social functioning, and long-term health outcomes and are associated with increased risks of substance use, self-harm, and psychiatric comorbidities later in life (6). Furthermore, the tripartite model of emotional disorders suggests that depression and anxiety share a common underlying dimension of general psychological distress, explaining their frequent coexistence during adolescence (7, 8).

In Saudi Arabia, increasing attention has been directed toward adolescent mental health. Findings from the Saudi National Mental Health Survey reported that 40.1% of Saudi youth aged 15–30 years experienced at least one lifetime mental disorder, while only 14.47% of affected individuals received treatment (9). Earlier school-based studies conducted in the Aseer Region also demonstrated high prevalence rates of depression, anxiety, and stress among secondary school students. A study among male students in Abha reported prevalence rates of 38.2% for depression, 48.9% for anxiety, and 35.5% for stress, whereas a subsequent study among female students reported even higher rates, particularly for anxiety and stress (10, 11).

Despite these findings, important evidence gaps remain. Most previous studies in the Aseer Region were conducted more than a decade ago, included only single-sex student populations, and did not employ multivariable analytical approaches to account for potential sociodemographic confounders. In addition, Saudi Arabia has undergone substantial educational and sociocultural changes in recent years, which may have influenced the mental health profile of adolescents. Consequently, updated epidemiological evidence based on mixed-sex student populations is needed to better understand the current burden and determinants of psychological distress in this setting.

Therefore, this study aimed to assess the prevalence and severity of depression, anxiety, and stress among male and female secondary school students in Abha City, Saudi Arabia, and to identify the independent sociodemographic and family-related factors associated with these psychological conditions using multivariable logistic regression analysis. This study provides updated epidemiological evidence from the Aseer Region that may support school-based mental health screening, guide targeted preventive interventions, and inform adolescent mental health policies in Saudi Arabia.

## 2 Methods

### 2.1 Study design and setting

A cross-sectional analytical study was conducted among secondary school students in Abha City, Aseer Region, Kingdom of Saudi Arabia, between October and December 2025. The study targeted governmental general secondary schools under the supervision of the Directorate of Education in Aseer Region. In the Saudi educational system, secondary school comprises Grades 10–12 and typically includes students aged 15–18 years. The study population included all male and female students enrolled during the academic year 1446–1447 H (corresponding to the 2025–2026 academic year).

Eligible participants were students aged 15–18 years who were enrolled in one of the selected governmental secondary schools, were present on the day of data collection, and were able to complete the questionnaire independently. Students with a previous physician-diagnosed psychiatric disorder were excluded to minimize potential confounding. Students who declined participation, were absent during data collection, or submitted substantially incomplete questionnaires were also excluded from the final analysis.

### 2.2 Sample size calculation

The required sample size was calculated using the standard formula for estimating a population proportion in cross-sectional studies (12):


$$n=\frac{Z^{2}\times P\left(1-P\right)}{d^{2}}$$

Assuming a 95% confidence level (Z = 1.96), an expected prevalence of 50%, and a margin of error of 5%, the minimum estimated sample size was 384 students. To compensate for possible non-response and incomplete questionnaires, the target sample size was increased to 400 students.

### 2.3 Sampling and data collection approach

A multistage stratified random sampling technique was employed. Governmental secondary schools in Abha City were first stratified according to students’ sex because schools are administered separately for males and females in Saudi Arabia. Two boys’ schools and two girls’ schools were then selected using simple random sampling from the official list of eligible governmental secondary schools provided by the Directorate of Education.

Within each selected school, one class was randomly selected from each of Grades 10, 11, and 12 using simple random sampling. All students in the selected classes were invited to participate until the predetermined sample size was achieved. Equal numbers of students were recruited from the selected schools to obtain the final study sample of 400 participants. The equal distribution of male (n = 200) and female (n = 200) students reflected the sampling strategy across sex-stratified schools rather than *post hoc* matching.

Data were collected during regular school hours using anonymous self-administered questionnaires. Standardized instructions regarding the study objectives and questionnaire completion were provided by the researcher and trained school staff. Participation was voluntary, and students were informed that they could withdraw at any stage without consequences.

A pilot study involving 30 students from schools not included in the final sample was conducted to assess the clarity, comprehensibility, feasibility, and estimated completion time of the questionnaire. Minor wording modifications were made to improve clarity, while no changes were required to the questionnaire content or study procedures. Pilot participants were excluded from the final analysis.

### 2.4 Study tools

Data were collected using a structured questionnaire consisting of two sections. The first section collected sociodemographic and family-related information, including age, sex, smoking status, chronic disease, parental educational level, parental occupation, family size, parental marital status, and family history of psychiatric disorders.

The second section consisted of the validated Arabic version of the Depression, Anxiety, and Stress Scale (DASS-42) (13). The DASS-42 is a validated self-administered instrument comprising 42 items equally distributed across three subscales measuring depression, anxiety, and stress (14 items each). Each item is rated on a four-point Likert scale (0–3), with higher scores indicating greater symptom severity. Subscale scores were summed separately and classified into normal, mild, moderate, and severe/extremely severe categories according to the original DASS-42 scoring guidelines (13). Detailed scoring criteria and cut-off values are provided in **Supplementary Material 1**.

The Arabic version of the DASS-42 has demonstrated satisfactory validity and reliability in Arabic-speaking populations and has been widely used in adolescent mental health research (11).

### 2.5 Study variables

The dependent variables were the presence and severity of depression, anxiety, and stress symptoms measured using the DASS-42. Independent variables included age group (15 years, 16 years, and 17–18 years), sex, smoking status, chronic disease, family size, parental educational level, parental occupation, parental marital status, and family history of psychiatric disorders.

### 2.6 Statistical analysis

Data management and statistical analyses were conducted using R statistical software version 4.4.3 (R Foundation for Statistical Computing, Vienna, Austria). Data were reviewed for completeness, consistency, and coding accuracy before analysis. Descriptive statistics were used to summarize participant characteristics. Categorical variables were presented as frequencies and percentages. Associations between categorical independent variables and depression, anxiety, and stress were evaluated using Pearson’s chi-square test or Fisher’s exact test, as appropriate. Variables that were statistically significant in the bivariate analyses were considered for inclusion in the multivariable logistic regression models. Smoking status was additionally retained in all models as an *a priori* clinically relevant variable because of its well-established association with depression, anxiety, and stress among adolescents, regardless of its statistical significance in the bivariate analyses. Variables that did not contribute significantly to the final models were removed during the model-building process. Adjusted odds ratios (AORs) with corresponding 95% confidence intervals (CIs) were reported for variables retained in the final models.

Multivariable logistic regression was selected because the study outcomes were analyzed as binary variables (presence versus absence of symptoms), allowing simultaneous adjustment for multiple sociodemographic and family-related factors. For multivariable logistic regression analyses, parental educational level was dichotomized into lower (illiterate, primary, and intermediate) and higher (secondary, university, and postgraduate) educational levels. Parental marital status was also dichotomized into living together and not living together (divorced or one deceased parent). Age group and family size were entered into the regression models as ordinal variables to estimate the change in odds associated with each incremental increase in category, whereas parental occupation and the remaining categorical variables were entered as indicator (dummy) variables. Adjusted odds ratios (AORs) with 95% confidence intervals (CIs) were calculated to estimate the strength of independent associations. Variables not retained in the final multivariable model for a specific outcome are indicated by a dash (—) in **Table 1**. A two-sided p-value<0.05 was considered statistically significant. To facilitate interpretation of the multivariable findings, the adjusted odds ratios and corresponding 95% confidence intervals were additionally presented graphically using a forest plot.

**Table 1:** Multivariable logistic regression analysis of factors associated with depression, anxiety, and stress among secondary school students (n = 400).

Variable	Category (Reference)	Depression AOR (95% CI)	p-value	Anxiety AOR (95% CI)	p-value	Stress AOR (95% CI)	p-value
Age (years)	Increasing age (Ref: 15 years)	1.19 (0.69–2.06)	0.519	—	—	—	—
Sex	Female (Ref: Male)	3.60 (1.68–7.72)	**<0.001**	2.95 (1.55–5.62)	**<0.001**	3.08 (1.57–6.03)	**<0.001**
Smoking status	Smoker (Ref: Non-smoker)	3.80 (1.71–8.44)	**<0.001**	3.75 (1.71–8.22)	**<0.001**	3.45 (1.55–7.65)	**0.002**
Chronic disease	Yes (Ref: No)	6.62 (2.15–20.39)	**<0.001**	—	—	3.37 (1.32–8.58)	**0.011**
Family size (members)	Increasing family size (Ref:<5 members)	0.71 (0.41–1.21)	0.210	—	—	0.46 (0.26–0.80)	**0.006**
Family history of psychiatric disorders	Yes (Ref: No)	4.90 (1.79–13.41)	**0.002**	—	—	5.46 (1.99–15.01)	**0.001**
Father’s education	Higher (Ref: Lower)	0.82 (0.65–1.03)	0.092	0.87 (0.70–1.09)	0.292	—	—
Mother’s education	Higher (Ref: Lower)	0.88 (0.69–1.12)	0.320	0.78 (0.61–1.01)	0.055	—	—
Father’s occupation	Military (Ref: Governmental)	1.12 (0.75–1.68)	0.578	1.04 (0.71–1.44)	0.820	1.15 (0.78–1.72)	0.490
	Private sector (Ref: Governmental)	0.95 (0.60–1.50)	0.823	0.97 (0.64–1.47)	0.920	0.91 (0.58–1.42)	0.680
	Retired (Ref: Governmental)	1.35 (0.65–2.80)	0.418	1.40 (0.69–2.91)	0.315	1.48 (0.75–3.10)	0.250
	Other (Ref: Governmental)	0.88 (0.35–2.21)	0.785	0.91 (0.39–2.28)	0.850	0.85 (0.32–2.15)	0.740
Mother’s employment	Employed (Ref: Unemployed)	0.61 (0.32–1.19)	0.157	2.22 (1.19–4.14)	**0.011**	—	—
Parental marital status	Not living together (Ref: Living together)	1.50 (1.02–2.22)	**0.041**	—	—	1.30 (0.90–1.88)	0.155

AOR, adjusted odds ratio; CI, confidence interval; Ref, reference category. Adjusted odds ratios (AORs) with corresponding 95% confidence intervals (CIs) were estimated using separate multivariable logistic regression models for depression, anxiety, and stress. Reference categories are indicated in parentheses. For ordinal variables (age and family size), the AOR represents the change in odds associated with each incremental increase in category. Lower parental educational level included illiterate, primary, and intermediate education, whereas higher educational level included secondary, university, and postgraduate education. Governmental occupation was selected as the reference category because it represented the largest occupational group in the study population. Parental marital disruption refers to divorced parents or one deceased parent. Smoking status was retained in all multivariable models as an a priori clinically relevant variable despite not reaching statistical significance in the corresponding bivariate analyses. Variables not retained in the final multivariable model for a specific outcome are indicated by a dash (—). Statistically significant associations (p< 0.05) are shown in bold.

### 2.7 Ethical considerations

Ethical approval was obtained from the Institutional Review Board of the Ministry of Health, Aseer Region, Saudi Arabia (IRB Registration No. H-06-B-091; Approval No. IRB-133-2025). Additional administrative approval was obtained from the Directorate of Education in Abha City and the principals of the participating secondary schools before data collection. Because the study involved adolescents younger than 18 years, written informed consent was obtained from parents or legal guardians, and written assent was obtained from the participating students in accordance with the requirements of the Institutional Review Board and local regulations. Students aged 18 years provided their own written informed consent. Participation was entirely voluntary, and all participants were informed about the study objectives, procedures, and their right to decline participation or withdraw at any time without any academic or personal consequences. To ensure confidentiality and anonymity, questionnaires did not contain personally identifiable information, and all collected data were securely stored with access restricted to the research team. The study was conducted in accordance with the ethical principles of the Declaration of Helsinki and the research regulations of the Ministry of Health, Saudi Arabia.

## 3 Results

### 3.1 Sociodemographic and family characteristics

Of the students invited to participate, 400 met the eligibility criteria, completed the questionnaire, and were included in the final analysis (**Table 2**). The study sample comprised an equal number of male and female students (50.0% each). Most participants were 16 years old (80.5%), while 11.5% were aged 15 years and 8.0% were aged 17–18 years. Smoking was reported by 5.8% of students, and 5.5% had a chronic disease. The majority of participants belonged to families comprising 5–10 members (79.8%). Regarding parental educational attainment, university education was the most common level among fathers (44.0%), whereas mothers most frequently had university (26.5%) or secondary (24.0%) education. Governmental employment was the most common paternal occupation (43.0%), while most mothers were unemployed (67.0%). A positive family history of psychiatric disorders was reported by 5.5% of participants, and 91.3% reported that their parents were living together.

**Table 2:** Sociodemographic and family characteristics of the study participants and their association with depressive symptoms (n = 400).

Variable	Category	Total n (%)	Depression absent n (%)	Depression present n (%)	p-value
Age group (years)	15	46 (11.5)	36 (78.3)	10 (21.7)	0.035
	16	322 (80.5)	188 (58.4)	134 (41.6)	
	17–18	32 (8.0)	20 (62.5)	12 (37.5)	
Sex	Male	200 (50.0)	142 (71.0)	58 (29.0)	<0.001
	Female	200 (50.0)	102 (51.0)	98 (49.0)	
Smoking status	Smoker	23 (5.8)	10 (43.5)	13 (56.5)	0.076
	Non-smoker	377 (94.3)	234 (62.1)	143 (37.9)	
Chronic disease	Yes	22 (5.5)	6 (27.3)	16 (72.7)	0.001
	No	378 (94.5)	238 (63.0)	140 (37.0)	
Family size (members)	<5	22 (5.5)	5 (22.7)	17 (77.3)	<0.001
	5–10	319 (79.8)	208 (65.2)	111 (34.8)	
	>10	59 (14.8)	31 (52.5)	28 (47.5)	
Father’s education	Illiterate	12 (3.0)	5 (41.7)	7 (58.3)	0.002
	Primary	29 (7.3)	16 (55.2)	13 (44.8)	
	Intermediate	35 (8.8)	11 (31.4)	24 (68.6)	
	Secondary	74 (18.5)	46 (62.2)	28 (37.8)	
	University	176 (44.0)	115 (65.3)	61 (34.7)	
	Postgraduate	74 (18.5)	51 (68.9)	23 (31.1)	
Mother’s education	Illiterate	47 (11.8)	24 (51.1)	23 (48.9)	0.020
	Primary	67 (16.8)	33 (49.3)	34 (50.7)	
	Intermediate	47 (11.8)	25 (53.2)	22 (46.8)	
	Secondary	96 (24.0)	69 (71.9)	27 (28.1)	
	University	106 (26.5)	67 (63.2)	39 (36.8)	
	Postgraduate	37 (9.3)	26 (70.3)	11 (29.7)	
Father’s occupation	Military	53 (13.3)	19 (35.8)	34 (64.2)	<0.001
	Governmental	172 (43.0)	126 (73.3)	46 (26.7)	
	Private sector	135 (33.8)	80 (59.3)	55 (40.7)	
	Retired	25 (6.3)	9 (36.0)	16 (64.0)	
	Other	15 (3.8)	10 (66.7)	5 (33.3)	
Mother’s employment status	Unemployed	268 (67.0)	151 (56.3)	117 (43.7)	0.007
	Employed	132 (33.0)	93 (70.5)	39 (29.5)	
Family history of psychiatric disorders	Yes	22 (5.5)	7 (31.8)	15 (68.2)	0.004
	No	378 (94.5)	237 (62.7)	141 (37.3)	
Parental marital status	Living together	365 (91.3)	232 (63.6)	133 (36.4)	0.002
	Divorced	16 (4.0)	7 (43.8)	9 (56.3)	
	One parent deceased	19 (4.8)	5 (26.3)	14 (73.7)	

Values are presented as number (percentage). Percentages in the Total column were calculated using the overall study population (N = 400) (column percentages), whereas Percentages in the Depression absent and Depression present columns were calculated within each category (row percentages). P-values were calculated using Pearson’s chi-square test or Fisher’s exact test, as appropriate. Statistical significance was set at p< 0.05.

### 3.2 Prevalence and severity of negative emotional states

**Figure 1:**
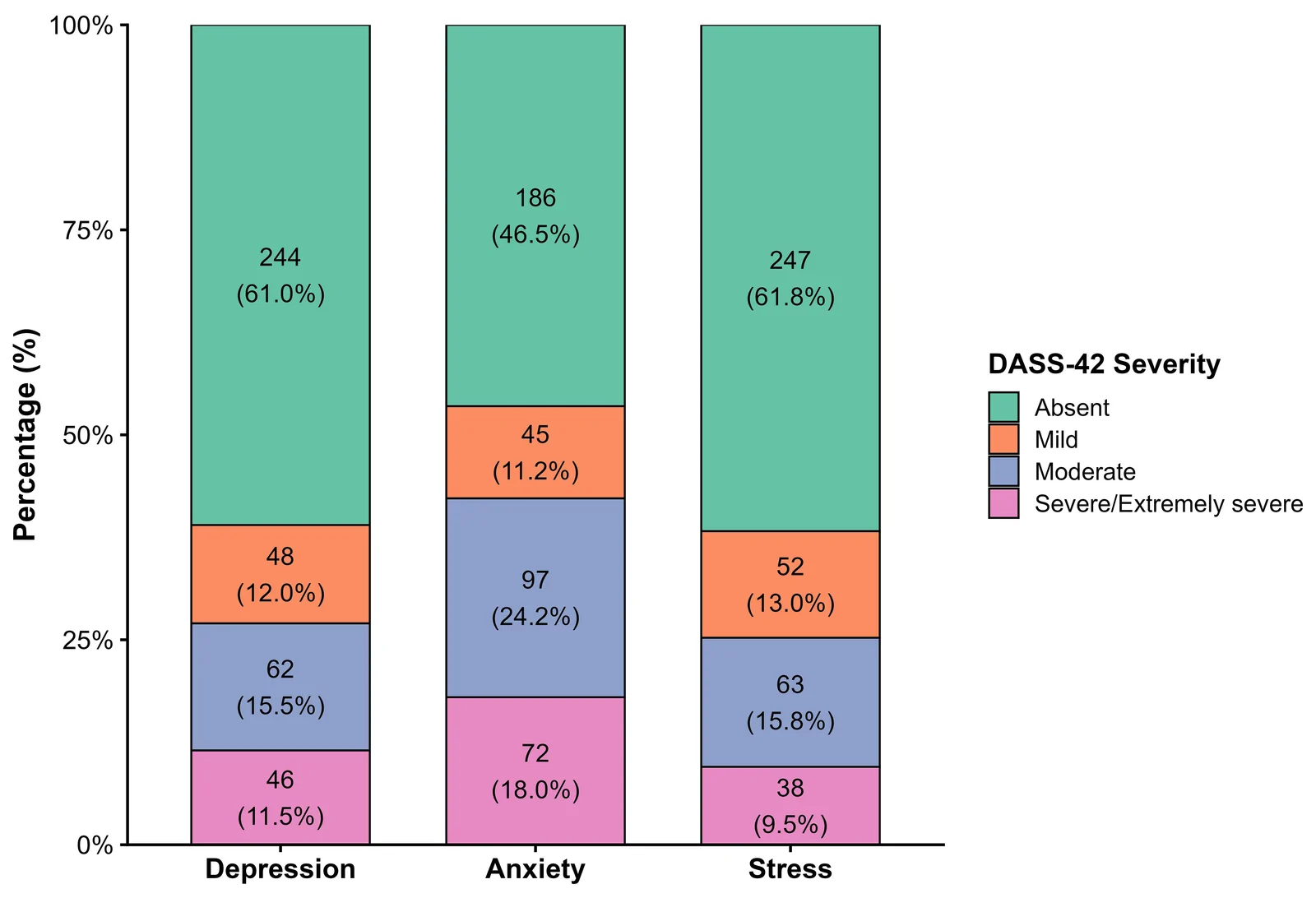
Distribution of depression, anxiety, and stress severity according to the DASS-42 among secondary school students (n = 400). Bars represent the percentage of students within each severity category. Values inside each segment indicate the number of participants and corresponding percentage.

Overall, depressive symptoms were identified in 39.0% of students, anxiety symptoms in 53.5%, and stress symptoms in 38.3%, with anxiety representing the most prevalent psychological condition, followed by depression and stress. Moderate severity was the most common symptomatic category for all three psychological outcomes, affecting 15.5% of students for depression, 24.2% for anxiety, and 15.8% for stress. Severe or extremely severe symptoms were reported by 11.5%, 18.0%, and 9.5% of students for depression, anxiety, and stress, respectively. **Figure 1** illustrates the distribution of symptom severity across the three psychological outcomes.

### 3.3 Bivariate analysis of factors associated with negative emotional states

The prevalence of depressive symptoms differed significantly according to several sociodemographic and family-related factors (**Table 2**). Female students had a significantly higher prevalence of depressive symptoms than male students (49.0% vs. 29.0%, p< 0.001). Students with chronic diseases also had a higher prevalence of depressive symptoms than those without chronic diseases (72.7% vs. 37.0%, p = 0.001). Depressive symptoms were more common among students from smaller families (<5 members) (77.3%, p< 0.001). Significant associations were also observed with both fathers’ and mothers’ educational levels (p = 0.002 and p = 0.020, respectively). The highest prevalence of depressive symptoms was observed among students whose fathers were military personnel (64.2%) or retired (64.0%) (p< 0.001). Depression was also more prevalent among students with a positive family history of psychiatric disorders (68.2% vs. 37.3%, p = 0.004), among those whose parents were not living together, particularly when one parent was deceased (73.7%, p = 0.002), and among students whose mothers were unemployed (43.7% vs. 29.5%, p = 0.007).

**Table 3** shows that anxiety symptoms were significantly associated with sex, parental educational level, fathers’ occupation, and mothers’ employment status. No statistically significant association was observed between age group and anxiety symptoms (p = 0.232), although the highest prevalence was observed among 15-year-old students (65.2%). Female students had a significantly higher prevalence of anxiety than male students (68.0% vs. 39.0%, p< 0.001). No statistically significant associations were observed between anxiety symptoms and smoking status, chronic disease, family size, family history of psychiatric disorders, or parental marital status (all p > 0.05). In contrast, significant associations were identified with both paternal and maternal educational levels (p = 0.004 and p< 0.001, respectively). Anxiety prevalence was highest among students whose fathers had postgraduate education (68.9%) and whose mothers had postgraduate education (86.5%). Significant differences were also observed according to fathers’ occupation (p = 0.002), with the highest prevalence among students whose fathers were retired (84.0%). Furthermore, anxiety symptoms were significantly more prevalent among students whose mothers were employed than among those whose mothers were unemployed (62.9% vs. 48.9%, p = 0.008).

**Table 3:** Prevalence of anxiety among secondary school students according to sociodemographic and family characteristics (n = 400).

Variable	Category	Anxiety absent n (%)	Anxiety present n (%)	p-value
Age group (years)	15	16 (34.8)	30 (65.2)	0.232
	16	154 (47.8)	168 (52.2)	
	17–18	16 (50.0)	16 (50.0)	
Sex	Male	122 (61.0)	78 (39.0)	<0.001
	Female	64 (32.0)	136 (68.0)	
Smoking status	Smoker	10 (43.5)	13 (56.5)	0.765
	Non-smoker	176 (46.7)	201 (53.3)	
Chronic disease	Yes	6 (27.3)	16 (72.7)	0.063
	No	180 (47.6)	198 (52.4)	
Family size (members)	<5	7 (31.8)	15 (68.2)	0.083
	5–10	145 (45.5)	174 (54.5)	
	>10	34 (57.6)	25 (42.4)	
Father’s education	Illiterate	5 (41.7)	7 (58.3)	0.004
	Primary	12 (41.4)	17 (58.6)	
	Intermediate	14 (40.0)	21 (60.0)	
	Secondary	31 (41.9)	43 (58.1)	
	University	101 (57.4)	75 (42.6)	
	Postgraduate	23 (31.1)	51 (68.9)	
Mother’s education	Illiterate	18 (38.3)	29 (61.7)	<0.001
	Primary	31 (46.3)	36 (53.7)	
	Intermediate	30 (63.8)	17 (36.2)	
	Secondary	51 (53.1)	45 (46.9)	
	University	51 (48.1)	55 (51.9)	
	Postgraduate	5 (13.5)	32 (86.5)	
Father’s occupation	Military	18 (34.0)	35 (66.0)	0.002
	Governmental	88 (51.2)	84 (48.8)	
	Private sector	66 (48.9)	69 (51.1)	
	Retired	4 (16.0)	21 (84.0)	
	Other	10 (66.7)	5 (33.3)	
Mother’s employment status	Unemployed	137 (51.1)	131 (48.9)	0.008
	Employed	49 (37.1)	83 (62.9)	
Family history of psychiatric disorders	Yes	6 (27.3)	16 (72.7)	0.063
	No	180 (47.6)	198 (52.4)	
Parental marital status	Living together	169 (46.3)	196 (53.7)	0.307
	Divorced	10 (62.5)	6 (37.5)	
	One parent deceased	7 (36.8)	12 (63.2)	

Values are presented as number (percentage). Percentages in the Absent and Present columns are calculated within each category (row percentages). P-values were calculated using Pearson’s chi-square test or Fisher’s exact test, as appropriate. Statistical significance was set at p< 0.05.

**Table 4** presents the bivariate associations between participant characteristics and stress symptoms. Significant associations were observed with sex, chronic disease, family size, fathers’ occupation, family history of psychiatric disorders, and parental marital status. Female students had a significantly higher prevalence of stress symptoms than male students (50.0% vs. 26.5%, p< 0.001). Students with chronic diseases were also more likely to report stress symptoms than those without chronic diseases (59.1% vs. 37.0%, p = 0.039). Stress prevalence differed significantly according to family size (p = 0.009), with the highest prevalence observed among students from families with fewer than five members (68.2%). Significant differences were also observed according to fathers’ occupation (p< 0.001), with the highest prevalence among students whose fathers were retired (76.0%). Furthermore, students with a positive family history of psychiatric disorders had a significantly higher prevalence of stress symptoms than those without such a history (68.2% vs. 36.5%, p = 0.003). Stress prevalence also varied significantly according to parental marital status (p = 0.019), with the highest prevalence observed among students with one deceased parent (68.4%). No statistically significant associations were found between stress symptoms and age group, smoking status, fathers’ education, mothers’ education, or mothers’ employment status (all p > 0.05).

**Table 4:** Prevalence of stress among secondary school students according to sociodemographic and family characteristics (n = 400).

Variable	Category	Stress absent n (%)	Stress present n (%)	p-value
Age group (years)	15	28 (60.9)	18 (39.1)	0.989
	16	199 (61.8)	123 (38.2)	
	17–18	20 (62.5)	12 (37.5)	
Sex	Male	147 (73.5)	53 (26.5)	<0.001
	Female	100 (50.0)	100 (50.0)	
Smoking status	Smoker	11 (47.8)	12 (52.2)	0.157
	Non-smoker	236 (62.6)	141 (37.4)	
Chronic disease	Yes	9 (40.9)	13 (59.1)	0.039
	No	238 (63.0)	140 (37.0)	
Family size (members)	<5	7 (31.8)	15 (68.2)	0.009
	5–10	200 (62.7)	119 (37.3)	
	>10	40 (67.8)	19 (32.2)	
Father’s education	Illiterate	6 (50.0)	6 (50.0)	0.743
	Primary	18 (62.1)	11 (37.9)	
	Intermediate	18 (51.4)	17 (48.6)	
	Secondary	46 (62.2)	28 (37.8)	
	University	111 (63.1)	65 (36.9)	
	Postgraduate	48 (64.9)	26 (35.1)	
Mother’s education	Illiterate	31 (66.0)	16 (34.0)	0.281
	Primary	35 (52.2)	32 (47.8)	
	Intermediate	28 (59.6)	19 (40.4)	
	Secondary	61 (63.5)	35 (36.5)	
	University	64 (60.4)	42 (39.6)	
	Postgraduate	28 (75.7)	9 (24.3)	
Father’s occupation	Military	26 (49.1)	27 (50.9)	<0.001
	Governmental	115 (66.9)	57 (33.1)	
	Private sector	90 (66.7)	45 (33.3)	
	Retired	6 (24.0)	19 (76.0)	
	Other	10 (66.7)	5 (33.3)	
Mother’s employment status	Unemployed	158 (59.0)	110 (41.0)	0.101
	Employed	89 (67.4)	43 (32.6)	
Family history of psychiatric disorders	Yes	7 (31.8)	15 (68.2)	0.003
	No	240 (63.5)	138 (36.5)	
Parental marital status	Living together	230 (63.0)	135 (37.0)	0.019
	Divorced	11 (68.8)	5 (31.3)	
	One parent deceased	6 (31.6)	13 (68.4)	

Values are presented as number (percentage). Percentages in the Stress absent and Stress present columns were calculated within each category (row percentages). P-values were calculated using Pearson’s chi-square test or Fisher’s exact test, as appropriate. Statistical significance was set at p< 0.05.

### 3.4 Multivariable logistic regression analysis

Multivariable logistic regression analysis identified several independent factors associated with depression, anxiety, and stress (**Table 1**). Female sex was independently associated with all three psychological outcomes, including depression (AOR = 3.60, 95% CI: 1.68–7.72, *p* < 0.001), anxiety (AOR = 2.95, 95% CI: 1.55–5.62, *p* < 0.001), and stress (AOR = 3.08, 95% CI: 1.57–6.03, *p* < 0.001). Smoking status, which was retained in the multivariable models as an *a priori* clinically relevant variable, was independently associated with increased odds of depression (AOR = 3.80, 95% CI: 1.71–8.44, *p* < 0.001), anxiety (AOR = 3.75, 95% CI: 1.71–8.22, *p* < 0.001), and stress (AOR = 3.45, 95% CI: 1.55–7.65, *p* = 0.002). Students with chronic diseases had significantly higher odds of depression (AOR = 6.62, 95% CI: 2.15–20.39, *p* < 0.001) and stress (AOR = 3.37, 95% CI: 1.32–8.58, *p* = 0.011). Increasing family size was independently associated with lower odds of stress (AOR = 0.46, 95% CI: 0.26–0.80, *p* = 0.006). A positive family history of psychiatric disorders was independently associated with increased odds of depression (AOR = 4.90, 95% CI: 1.79–13.41, *p* = 0.002) and stress (AOR = 5.46, 95% CI: 1.99–15.01, *p* = 0.001). Maternal employment was independently associated with higher odds of anxiety (AOR = 2.22, 95% CI: 1.19–4.14, *p* = 0.011), whereas parental marital disruption was independently associated with increased odds of depression (AOR = 1.50, 95% CI: 1.02–2.22, *p* = 0.041).

**Figure 2:**
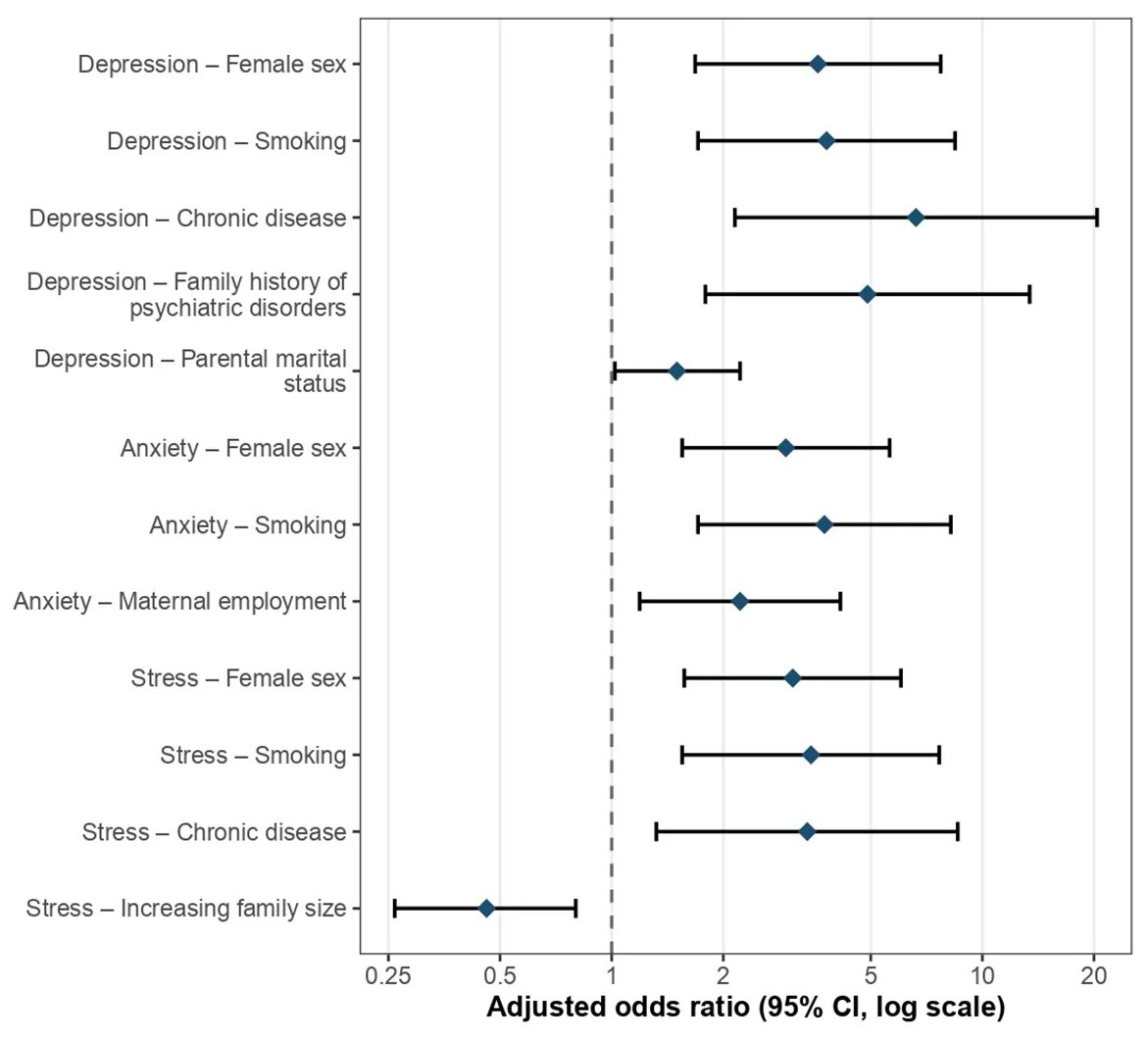
Forest plot showing adjusted odds ratios (AORs) and 95% confidence intervals (CIs) for variables retained in the final multivariable logistic regression models of depression, anxiety, and stress among secondary school students. Squares represent adjusted odds ratios, horizontal lines represent 95% confidence intervals, and the vertical dashed line denotes the null value (AOR = 1.0). Estimates to the right of the reference line indicate increased odds, whereas estimates to the left indicate decreased odds. Smoking status was retained in all models as an a priori clinically relevant variable.

Female sex and smoking were consistently associated with increased odds of depression, anxiety, and stress. Chronic disease and a positive family history of psychiatric disorders were independently associated with depression and stress, whereas maternal employment was associated only with anxiety. In contrast, Increasing family size was associated with lower odds of stress, while parental marital disruption was independently associated with depression. The magnitude and precision of these associations, expressed as adjusted odds ratios (AORs) with corresponding 95% confidence intervals (CIs), are presented graphically in **Figure 2**.

## 4 Discussion

The present study demonstrated a high prevalence of negative emotional states among secondary school students in Abha City, with anxiety representing the most common condition, followed by depression and stress. More importantly, a considerable proportion of students experienced moderate to severe symptom levels, indicating psychologically meaningful symptom burden rather than transient emotional fluctuations. These findings further highlight adolescent mental health as an important public health concern and emphasize the need for early identification and school-based psychological support programs.

Importantly, this study directly addresses a significant methodological and temporal gap in the existing literature regarding adolescent mental health in the southern regions of Saudi Arabia. While foundational studies conducted in the Aseer Region over a decade ago provided initial evidence of psychological distress, they were primarily restricted to single-sex populations and lacked robust adjustment for contemporary sociodemographic confounders (10, 11). By evaluating a recent, mixed-sex cohort and employing multivariable logistic regression modeling, the current research provides an updated and comprehensive epidemiological profile of adolescent mental health in the Aseer Region, offering contemporary evidence from southern Saudi Arabia (14). This approach allowed for the isolation of independent risk factors, thereby bridging the historical data gap with highly relevant, contemporary evidence.

The prevalence estimates observed in the present study are generally consistent with findings from regional and international studies reporting substantial levels of depression, anxiety, and stress among adolescents (15–17). In the current study, anxiety was the most prevalent psychological outcome, affecting 53.5% of students, followed by depression (39.0%) and stress (38.3%). Similar studies among secondary school students in Saudi Arabia and other countries have reported depression prevalence ranging from 30% to 43% (18, 19). The predominance of anxiety observed in our study is consistent with accumulating evidence indicating that anxiety disorders are among the most common mental health problems during adolescence (16, 18, 19). Meta-analytic evidence has estimated a pooled prevalence of anxiety symptoms of approximately 31% among children and adolescents, although considerably higher estimates have been reported in school-based populations (20). The relatively high prevalence of psychological symptoms observed in the present study may reflect the cumulative effects of academic demands, uncertainty regarding future educational opportunities, social expectations, and other psychosocial stressors commonly experienced during adolescence (21).

Adolescence is a sensitive developmental period characterized by emotional instability, identity formation, and evolving social relationships (22). During this stage, adolescents often experience conflicts within family and school environments, increasing vulnerability to depression, anxiety, and stress. Previous studies have demonstrated that emotional dysregulation during adolescence negatively affects academic performance, cognitive functioning, and social relationships (19, 23). Furthermore, chronic psychological distress during adolescence has been associated with long-term adverse outcomes, including substance misuse, self-harm, and persistence of psychiatric disorders into adulthood (24, 25).

Female students in the present study exhibited significantly higher prevalence rates of depression, anxiety, and stress than their male counterparts. Moreover, female sex remained an independent predictor of all three psychological outcomes after adjustment for potential confounding factors, highlighting its robust association with adolescent psychological distress. These findings are consistent with studies from Oman, Jordan, the United Arab Emirates, and Lebanon, which similarly reported greater psychological vulnerability among adolescent girls (15–17). Several biological, hormonal, psychological, and sociocultural mechanisms may contribute to this disparity. Hormonal changes during adolescence, greater emotional reactivity, interpersonal stress, and societal expectations may increase the susceptibility of female adolescents to depression, anxiety, and stress (26). In addition, girls may be more likely to internalize emotional distress and experience higher levels of academic and social pressure, further contributing to the observed sex differences in mental health outcomes.

The present study identified several important family- and health-related determinants of psychological distress. After adjustment for potential confounding factors, chronic disease remained independently associated with significantly higher odds of both depression and stress, while a positive family history of psychiatric disorders markedly increased the likelihood of these two psychological outcomes. Furthermore, parental marital disruption was independently associated with depressive symptoms, whereas increasing family size was associated with lower odds of stress, suggesting a potential protective effect of stronger family support and social connectedness. These findings reinforce previous evidence highlighting the central role of family environment, emotional support, and health status in shaping adolescent mental health (27–29). Adolescents exposed to family instability, hereditary vulnerability to psychiatric disorders, or chronic health conditions may face greater emotional challenges that increase their susceptibility to depression and stress (30).

Smoking also emerged as one of the strongest independent predictors of psychological distress in the present study. Students who reported smoking had substantially higher odds of depression, anxiety, and stress than non-smokers, even after adjustment for sociodemographic and family-related factors. This finding is consistent with previous research demonstrating a bidirectional relationship between tobacco use and psychological disorders during adolescence. Psychological distress may increase the likelihood of smoking initiation as a maladaptive coping strategy, while nicotine dependence may further exacerbate anxiety and depressive symptoms through neurobiological and behavioral mechanisms. These findings emphasize the importance of integrating tobacco prevention and smoking cessation initiatives into adolescent mental health promotion programs (31, 32).

Although parental educational level and paternal occupation were associated with some psychological outcomes in the bivariate analyses, these associations were no longer statistically significant after adjustment for potential confounders. This finding suggests that the influence of these sociodemographic characteristics may be mediated through broader family, health, and behavioral factors rather than representing independent determinants of adolescent mental health (33). In contrast, maternal employment remained independently associated with anxiety symptoms, indicating that family structure and caregiving dynamics may influence adolescents’ psychological well-being and warrant further investigation.

The observed overlap between depression, anxiety, and stress is consistent with the tripartite model of emotional disorders, which proposes shared dimensions of negative affectivity across these syndromes (33, 34). Academic stress, peer-related difficulties, inadequate coping strategies, and family-related stressors may collectively contribute to the coexistence of these emotional problems during adolescence. Moreover, the substantial proportion of students experiencing moderate to severe symptom levels suggests that many adolescents may require comprehensive psychological assessment and timely intervention rather than preventive counseling alone (35).

The findings of this study have important implications for adolescent mental health services and school health policies in Saudi Arabia (36, 37). Routine mental health screening within schools may facilitate the early identification of students at risk for emotional disorders. School-based interventions focusing on resilience building, stress management, emotional regulation, psychological counseling, and smoking prevention or cessation may help reduce the burden of depression, anxiety, and stress among adolescents. Strengthening collaboration among schools, families, and healthcare providers is also essential to improve mental health awareness, facilitate early referral, and ensure timely access to appropriate psychological support.

### 4.1 Strengths, limitations, and future perspectives

This study has several strengths. It provides one of the most recent epidemiological assessments of depression, anxiety, and stress among secondary school students in the Aseer Region, addressing an important evidence gap since the previous regional studies were conducted more than a decade ago. The inclusion of both male and female students, the use of a multistage stratified random sampling approach, and the application of the validated Arabic version of the DASS-42 enhance the representativeness and methodological rigor of the study. Furthermore, the use of multivariable logistic regression enabled adjustment for multiple sociodemographic and family-related factors, allowing identification of independent predictors of psychological distress.

Despite these strengths, several limitations should be considered when interpreting the findings. First, the cross-sectional design precludes establishing causal relationships between the identified factors and depression, anxiety, and stress. Second, data were collected using self-administered questionnaires, making the findings susceptible to recall bias and social desirability bias. Third, the study was conducted exclusively in governmental secondary schools in Abha City, which may limit the generalizability of the findings to students attending private schools or adolescents in other regions of Saudi Arabia. Fourth, although smoking emerged as an independent predictor of psychological distress, the relatively small number of smokers resulted in wider confidence intervals; therefore, this association should be interpreted cautiously and confirmed in studies with larger numbers of smokers. Finally, although the DASS-42 is a well-validated instrument for assessing psychological symptoms, it is a screening rather than a diagnostic tool; consequently, the reported prevalence reflects symptom burden rather than clinically confirmed psychiatric disorders.

Future longitudinal and multicenter studies are warranted to establish temporal and causal relationships between identified risk factors and psychological outcomes. Including students from private schools and other regions of Saudi Arabia would improve the generalizability of future findings. In addition, studies incorporating structured clinical diagnostic interviews alongside standardized screening instruments would provide a more comprehensive assessment of adolescent mental health. Future intervention studies evaluating school-based mental health promotion programs, resilience-building strategies, smoking prevention initiatives, and family-centered psychological support are also recommended to determine effective approaches for reducing the burden of depression, anxiety, and stress among Saudi adolescents.

## 5 Conclusion

This study demonstrated a high prevalence of depression, anxiety, and stress among secondary school students in Abha City, with anxiety emerging as the most common psychological condition. Multivariable logistic regression identified female sex and smoking as consistent independent predictors of all three psychological outcomes, while chronic disease, a positive family history of psychiatric disorders, smaller family size, maternal employment, and parental marital disruption were independently associated with specific dimensions of psychological distress. These findings highlight the important influence of individual, family, and health-related factors on adolescent mental health and underscore the need for early identification of students at increased risk. School-based mental health screening, psychological counseling, resilience-building programs, and smoking prevention initiatives should be integrated into existing school health services to promote early intervention and improve psychological well-being. Furthermore, strengthening collaboration among schools, families, and healthcare providers may facilitate timely identification, referral, and support for adolescents experiencing emotional distress. By providing updated evidence from a mixed-sex cohort of secondary school students in the Aseer Region, this study contributes contemporary epidemiological data that may inform adolescent mental health policies and guide future longitudinal research in Saudi Arabia.

## Data Availability

The raw data supporting the conclusions of this article will be made available by the authors, without undue reservation.
