# Supplementary material for: Prevalence and risk factors of depression, anxiety, and stress among secondary school students in Abha city, Saudi Arabia
Source: Front Psychiatry. 2026 Aug 21;17:1885953. doi: 10.3389/fpsyt.2026.1885953 (PMC13543033; doi:10.3389/fpsyt.2026.1885953)
Supplement: Supplementary file 1 [file Table1.docx]

**Supplementary table S1.** DASS-42 scoring system and severity classification used in the study

| **Psychological domain** | **Number of items** | **Score range** | **Normal** | **Mild** | **Moderate** | **Severe/Extremely severe*** |
| --- | --- | --- | --- | --- | --- | --- |
| Depression | 14 | 0–42 | 0–9 | 10–13 | 14–20 | ≥21 |
| Anxiety | 14 | 0–42 | 0–7 | 8–9 | 10–14 | ≥15 |
| Stress | 14 | 0–42 | 0–14 | 15–18 | 19–25 | ≥26 |

**Notes**

- The Depression, Anxiety, and Stress Scale (DASS-42) consists of 42 self-reported items, equally distributed across three subscales (14 items each).
- Each item is scored on a 4-point Likert scale ranging from 0 ("Did not apply to me at all") to 3 ("Applied to me very much or most of the time"), reflecting participants' experiences during the previous week.
- Scores for each subscale are calculated by summing the responses to the corresponding 14 items.
- In the present study, participants with mild, moderate, severe, or extremely severe scores were classified as having the corresponding psychological outcome for regression analyses, whereas those with normal scores served as the reference group.
- For descriptive presentation in the main manuscript, the severe and extremely severe categories were combined into a single category ("Severe/Extremely severe") because of the relatively small number of participants in the extremely severe category.

**Reference**

Lovibond, S. H., & Lovibond, P. F. (1995). Depression Anxiety Stress Scales (DASS--21, DASS--42) [Database record]. APA PsycTests. <https://doi.org/10.1037/t01004-000>.
